# Editorial: The Future of Perceptual Illusions: From Phenomenology to Neuroscience

**DOI:** 10.3389/fnhum.2017.00009

**Published:** 2017-02-03

**Authors:** Adam Reeves, Baingio Pinna

**Affiliations:** ^1^Department of Psychology, Northeastern UniversityBoston, MA, USA; ^2^Department of Architecture, Design and Planning, University of SassariAlghero, Italy

**Keywords:** perceptual illusions, editorial, collection, models, theoretical, ecological and environmental phenomena

We have accepted a total of 42 articles for this special issue, which together have earned 80,000 views as of June 2, 2016, showing the popularity of illusions as a subject for both authors and readers. The papers address conceptual and perceptual illusions, typically using psychophysical, computational, or phenonomological methods, or some mixture of these. Narrowly defined, an illusion is a mismatch between the physical and the phenomenal world, and this definition encompasses the great majority of the articles. Somewhat more broadly defined, though, illusory perceptions may be based on ambiguous stimulus information, which may or may not count as mismatches. Examples are the famous duck/rabbit image in which perceptual selection of a part (and hence a “negative hallucination”—a destruction of the rest) occurs, rather than misperception of the whole; or in displays in which lightness, brightness, and illumination are potentially confounded, as considered by Rudd; or when local and global motions are intertwined, as in the paper by Erlikhman et al.

The papers fall into 5 rather broad categories: models and theory, illusions of geometry and position, illusions of motion, illusions of brightness and color, and multisensory and other illusions. We list them by author in this order to facilitate comparison on like themes, but here we wish to emphasize the broader point that the authors find that the study of illusions can be useful in most, perhaps all, of vision science. Indeed, taken together, the papers support what we claim to be the *fundamental principle of perceptual science*, namely, that *illusions (especially of the mismatch variety) illustrate basic processes in perception*; they should not be disregarded as unecological, accidental, or due to random failures of processing. The papers by Kingdom, and by Zeman et al., illustrate this point; Allard and Faubert specifically rely on basic visual mechanisms (not accidental effects) to explain particular illusions. We do have two caveats; first, as pointed out by Gibson, the emphasis on “what is inside the head” may ignore what is really in the world; and second, an over-reliance on the fundamental principle may limit research to variations on known process–illusion relations, restricting exploration of new phenomena—who could have predicted, for example, the now well-known rubber-hand illusion (c.f. Limanowski's study)? Nevertheless, the current papers, and the vast stream of background studies that they refer to, make a good case for the fundamental principle. A consequence is that brain science should only be invoked when the neural modeling is sophisticated enough (as in Grossberg's paper) to have some chance of accounting for the typically elaborate complications of the behavioral and phenomenological data; brain scanning is not enough. Our collection implicitly confirms this by emphasizing the psychological over the neural approaches to illusions, though this phase in the history of our science will surely pass as neural models develop and take hold.

## Models and theory

Carbon argues that in general, illusions specify the limits and capacity of our perceptual apparatus, in opposition to the ecological approach associated with Gibson.

Kingdom explains how the illusory dark and bright bars seen at the edges of a luminance trapezoid provide a test bed for models of brightness.

Domijan offers a new filling-in model to account for brightness illusions in which responses to low-contrast contours are enhanced by nearby collinear high-contrast contours.

Grossberg et al. shows how two visual illusions can arise from adaptive neural processes.

Laparra and Malo show that both aftereffects and nonlinear gain changes, due to adaptation to visual scenes, can be derived from Sequential Principal Curves Analysis (SPCA), a possible improvement over previous models in which these adaptation effects are treated separately.

Rudd discusses models of lightness which assume that the visual system first extracts local steps in log luminance.

Scocchia et al. argue that the world as it appears to the viewer is the result of a complex process of inference performed by the brain, a (to many) counter-intuitive assertion which can be illustrated with noisy, feeble, or ambiguous visual stimulation.

Zavagno et al. consider illusions from three classic viewpoints, Ecological, Cognitive, and Gestalt.

Zeman et al. show that the simultaneous contrast illusion can be explained, in 24 of 27 varieties (extraordinary how many there are !) by a set of novel filters whose kernels span multiple sizes and shapes, followed in a few cases by a second stage of normalization.

## Illusions of geometry and position

Bertamini explains why observers assume that Venus is admiring her own reflection in a mirror when she is actually looking at the reflection of the painter.

Gori et al. reviews how visual illusions may help us understand the visual deficits in developmental dyslexia and autism spectrum disorder, and perhaps develop new clinical tools as a result.

Morikawa et al. relate the eye size illusion induced by the eyebrows to the classic Delboeuf illusion and perhaps surprisingly argue they involve the same mechanism.

Pinna offers a solution of the apparent conflict between the Rectangle illusion and Helmholtz's Square illusion, whose geometrical effects are superficially opposed.

Shapiro et al. discuss how humans are able to maintain a relatively stable representation of objects and features even though the visual system processes many aspects of the world separately.

Strother et al. argue that a subtle illusory curvature, perceived when none exists, is an instance of a more general illusory curvature phenomenon.

Tseng et al. report a naturally occurring “windsurfer” illusion in which the small end of a sail appears to be pointed away from the observer even when it is closer.

Talasli and Inan show that the illusion reflects the operation of an interposition cue to depth followed by the application of Emmert's Law to the—now seen in different depth planes—occulded and occulding parts of the image, a theory that predicts a new illusion that they also demonstrate.

Safran et al. suggest that Bacon's paintings reflect a rare central perception disorder called dysmorphopsia, as supported by his descriptions of the dynamic perceptual distortions he sometimes experiences.

Yun et al. studied neural correlates accompanying the spiral illusion, and three variants derived from it, in a new approach.

## Illusions of motion

Allard and Faubert an illusion in which the perceived path of a moving textured target follows different paths when viewed foveally and peripherally can be explained by reduced position acuity with eccentricity, and does not imply different ways of processing central and peripheral motion.

Caniard et al. found that local motion produced stronger illusory displacements in the perceived position of globally static objects under *active* than under *passive* viewing. We are all reminded that the passive viewing of displays, so typical of experimentation today, can mislead.

Dürsteler illustrates various visual illusions created by two overlapping surfaces each defined by textures of independent visual features.

Erlikhman et al. discuss the perception of form, global motion, and continuous boundaries, caused by changes in local texture elements.

Mruczek et al. show that target motion, peripheral viewing, and smooth pursuit eye movements, all increase the Ebbinghaus illusion by adding (retinal) noise which leads to uncertainty about target size.

## Illusions of brightness and color

Coia and Crognale discuss how the watercolor illusion depends on the contrast of the two lines that create the illusion.

Devinck et al. discuss how such models may account in detail for Pinna's watercolor illusion.

Gilchrist discusses the problem, critical for modeling, of whether observers typically make brightness, brightness contrast, or lightness matches, and concludes they make lightness matches when instructed to do so.

Kimura and Kuroki discuss both assimilative and non-assimilative color spreading in the watercolor configuration composed of wavy double contours, depending on the luminance conditions of the inner and outer contours. They also discuss non-assimilative color spreading in what has hitherto been characterized as an assimilative effect.

Li et al. discuss a classic problem, why the retinal blind spot is rarely noticed the daily life.

Pereverzeva and Murray address for an illusion the classic problem that accurate perception of surface reflectance poses a significant computational problem for vision.

Roncato discusses the effect of contrast polarity on the integration or interpolation of fragmented edges into visual contours.

## Multisensory and other illusions

Bainbridge et al. report that their participants reported equally confident rating of the motions of illusory and real sounds, and could not distinguish them when tested objectively, a new illusion that may provide insight into the neural mechanisms of auditory spatial localization.

Blanuša and Zdravković find the H-V illusion is equal in perception and imagery, suggesting that these processes share a neural substrate, but also find an unexpected gender effect on the size of mental image for medium and large stimuli.

Dassonville and Reed, argue (rather subtly) that Perception and Action need not be separate systems, even though Perception is susceptible to illusions, while Action is not, because dissociations will occur for any illusion that is caused by a distortion of the observer's egocentric reference frame.

Kilteni et al. discuss “Body ownership illusions,” in which fake parts seems to be part of our own body, and how Bayesian causal inference begins to explain why and when these occur.

Limanowsk et al. discusses the possible meaning of the extraordinary rubber hand illusion, in which congruent touch of a hidden hand and a fake counterpart makes one feel that the fake hand is part of one's own body.

Marques et al. discuss the multi-sensory integration implied by the classic McGurk illusion, in which a visual stimulus /ga/ is paired with the auditory stimulus /ba/ and people hear /da/.

Vidal and Barrès investigate the perceptual stabilization effect of an additional sound on the dynamics of binocular rivalry.

In sum, we thank the authors for a splendid collection of very fine papers on this important topic.

## Author contributions

AR wrote the Editorial with the inspiration and guidance of BP.

### Conflict of interest statement

The authors declare that the research was conducted in the absence of any commercial or financial relationships that could be construed as a potential conflict of interest.

